# Simultaneous inferences based on empirical Bayes methods and false discovery rates ineQTL data analysis

**DOI:** 10.1186/1471-2164-14-S8-S8

**Published:** 2013-12-09

**Authors:** Arindom Chakraborty, Guanglong Jiang, Malaz Boustani, Yunlong Liu, Todd Skaar, Lang Li

**Affiliations:** 1 Department of Medical and Molecular Genetics, Indiana University School of Medicine, Indianapolis, Indiana 46202, USA; 2 Center for Computational Biology and Bioinformatics, Indiana University School of Medicine, Indianapolis, Indiana 46202, USA; 3 Regenstrief Institute, Indianapolis, Indiana 46202, USA; 4 Division of Clinical Pharmacology, Department of Medicine, Indiana University School of Medicine, Indianapolis, Indiana 46202, USA

## Abstract

**Background:**

Genome-wide association studies (GWAS) have identified hundreds of genetic variants associated with complex human diseases, clinical conditions and traits. Genetic mapping of expression quantitative trait loci (eQTLs) is providing us with novel functional effects of thousands of single nucleotide polymorphisms (SNPs). In a classical quantitative trail loci (QTL) mapping problem multiple tests are done to assess whether one trait is associated with a number of loci. In contrast to QTL studies, thousands of traits are measured alongwith thousands of gene expressions in an eQTL study. For such a study, a huge number of tests have to be performed ($\sim 10^{6}$). This extreme multiplicity gives rise to many computational and statistical problems. In this paper we have tried to address these issues using two closely related inferential approaches: an empirical Bayes method that bears the Bayesian flavor without having much *a priori *knowledge and the frequentist method of false discovery rates. A three-component t-mixture model has been used for the parametric empirical Bayes (PEB) method. Inferences have been obtained using Expectation/Conditional Maximization Either (ECME) algorithm. A simulation study has also been performed and has been compared with a nonparametric empirical Bayes (NPEB) alternative.

**Results:**

The results show that PEB has an edge over NPEB. The proposed methodology has been applied to human liver cohort (LHC) data. Our method enables to discover more significant SNPs with FDR<10% compared to the previous study done by Yang et al. (*Genome Research*, 2010).

**Conclusions:**

In contrast to previously available methods based on p-values, the empirical Bayes method uses local false discovery rate (lfdr) as the threshold. This method controls false positive rate.

## Introduction

Genome-wide association studies (GWASs) have done a remarkable progress in searching for susceptibility genes. In GWAS, instead of one gene at a time, variation across the entire genome is tested for association with disease risk. GWASs exploit the linkage disequilibrium (LD) relationships among single nucleotide polymorphisms (SNPs), making it possible to assay genome by testing a finite number of SNPs. Till date, the signals that can be discovered through GWAS has not been reported exhaustively. It is important to annotate SNPs information on expression for the better understanding of the genes and mechanisms driving the association. In many situations, there are more common variants truly associated with disease. These variants are highly likely to be expression quantitative trait loci (eQTLs). eQTLs are derived from polymorphisms in the genome that result in differential measurable transcript levels. Microarrays are used to measure gene expression levels across genetic mapping populations. For at least a subset of complex disorders, gene expression levels could be used as a surrogate/biomarker for classical phenotypes. The gene underlying the eQTL is considered to be an excellent candidate for phenotypic QTL.

eQTL mapping is a statistical technique to locate genomic intervals, that are likely to regulate the expression of each transcript, by correlating quantitative measurements of mRNA expression with genetic polymorphisms segregating in a population. In a GWAS, millions of SNPs are tested at once. Associations that initially appear to be significant must be statistically adjusted to account for the large number of tests being performed. A large number of false positives will result in if this correction is ignored. The multiple-testing correction, however, sets a very high threshold for genome-wide significance, on the order of $5\times 10^{-8}$ when a million SNPs are tested. In the vast majority cases, however, association studies have achieved only limited success. Large sample sizes are needed to achieve sufficient statistical power to detect risk alleles with effects weak enough to have escaped detection in the past; the disease risk alleles identified by GWASs so far do have weak effects, each with odds ratios of 1.1 or 1.2 [1].

Two closely related inferential procedures for multiple testing have been discussed in this work-afrequentist approach based on Benjamini and Hochberg's ([2]) false discovery rate procedure, and an empirical Bayes methodology developed in Efron et al. [3,4]. These two methods are not only very closely related, they can be used to support each other. In a classic two-sample problem in a microarray experiment, these approaches have been discussed by Efron and Tibshirani[5]. However, they have considered nonparametric empirical Bayes (NPEB) model. Parametric Bayesian modeling has been considered by Newton et al. [6], Lee et al. [7], Kendziroski et *al*. [8-10], Gelfond et *al*. [11]. Hierarchical models like gamma-gamma [6] or lognormal-normal [8] are used quite often in PEB procedures. These models suffer from a serious drawback that the variation is constant among genes. An extension has been done to these models by considering gene specific variations[12]. The application of empirical Bayes has been somehow not very common in literature. The obvious reason is that, experimenters have not brought us many data sets having the parallel structure necessary for empirical Bayes to do its stuff. Because of the recent surge in high-throughput ([13]) technologies and genome projects, many genome studies are now underway. These studies have become a major data generator in the post-genomics era. Empirical Bayes procedures seem to be particularly well-suited for combining information in expression data.

One of the fundamental statistical problems in microarray gene expression analysis is the need to reduce dimensionality of the transcripts. This can be achieved by identifying differentially expressed (DE) genes under different conditions or groups. Regulatory network can be obtained by associating differential expressions with the genotype of molecular markers. It is possible to have a large number of DE genes that influences a certain phenotype while their relative proportion is very small. It is very important to identify these DE genes from among the number of recorded genes [6,7,9,14,15]. Empirical Bayes methods provide a natural approach to reduce the dimensionality significantly [16,17]. Following the empirical Bayes approach DE genes are identified using the posterior probability for differential expression. EB approaches detect a DE gene by sharing information across the whole genome.

The development of the empirical Bayes methodologies that improve the power to detect DE genes essentially reduces to the choice of whether gene-specific effects should be modeled as fixed or random [18]. Both mean and error variance can be of either of these two: fixed or random. Fixed mean and random error variance has been considered by Wright and Simon [19] and Cui et al. [20] whereas Lonnstedt et al. [21], Tai and Speed [22], Lonnstedt and Speed [23] have considered both the parameters to be random. Random mean effect with homogeneous fixed error variance has been considered by Newton et al. [6,24], Kendziroski et al. [9] and Kendziroski et al. [10]. However an extension to this fixed error variance has been considered by Gelfond et al. [11]. They have considered discrete uniform prior for the variance component.

The paper is organized as follows. In the Methods section we introduce the necessary notations for our additive genetic model along with the notions of false discovery rate (fdr). In this section we have tried to establish the relationship between fdr and empirical Bayes. Methods section also describes, the proposed Expectation/Conditional Maximization Either (ECME) (Liu and Rubin [25]) in details. This algorithm generalizes the Expectation-Maximization algorithm with better convergence rate. A simulation study has been performed and described in the Results section. We show that proposed parametric empirical Bayes performs better compared to nonparametric empirical Bayes in terms of controlled fdr. In the Results section, as an application, we have applied the proposed methodology to the Liver Cohort (LHC) dataset. We conclude the article the Discussion section.

## Methods

In a microarray experiment, we obtain several thousand expression values, one or many for each gene. These studies offer an unprecedented ability to do large-scale studies of gene expression. Let us define *G_i_i *= 1*.....l *as the genomic marker(i.e. SNP), and *T_j_*(*j *= 1*......J*) as the transcripts. The identified eQTLs refer to the significant Gs that are associated with Ts. These associations can be found using a test statistics based on all  n samples. The genetic model for this association can be one of the three models: dominant, recessive and additive. Under the dominance model, we can have two genotypes for each of the SNPs. However for an additive model, three genotype groups are available. A transcript $T_{j}$ is assumed to be associated with marker $G_{i}$ if the mean level of expression of transcript $T_{j}$ for one genotype group is different from that of the other genotype group corresponding to that marker. Let $\mu_{T,G}^{\left(1\right)}$ and $\mu_{T,G}^{\left(0\right)}$ be the group means corresponding to the genotypes $G_{i}$. To test the hypothesis $H_{0}:\mu_{T,G}^{\left(1\right)}=\mu_{T,G}^{\left(0\right)}$, a few test statistics are proposed for microarray data analysis[26]. The present work is based on the statistic proposed by Efron et *al*. [4]. The test statistic is defined as

(1) $$Z_{ij}=\frac{{\bar{x}}_{T,G}^{\left(1\right)}-{\bar{x}}_{T,G}^{\left(0\right)}}{\left(a_{0i}+S_{ij}\right)}$$

where $S_{ij}$ is the usual standard deviations and $a_{0i}$ is defined to minimize the difference in the coefficient of variation of $Z_{ij}$ within classes of genes with approximately equal variance. A drawback of calculating $a_{0i}$ is the computational cost. Note that if $a_{0i}=0$, this reduces to usual t-statistic. Here $a_{0i}$ is considered to be 90^th ^percentile of all $S_{ij}$ values (Efron el *al*. [4]).

When expression measurements between two groups are compared for any transcript, the observations are partitioned into two user defined groups of sizes $n_{1}$ and $n_{2}$ with $n_{1}+n_{2}=n$. If there is no significant difference between the group means, the transcript is assumed to be equivalently expressed (EE). On the contrary, if significant difference is observed, the transcript is termed as differentially expressed (DE). For any transcript $T_{j}$ and SNP $G_{i}$ it may be either of these two: DE or EE. This uncertainty can be modeled by a mixture of two distributions as follows:

(2) $$f\left(Z_{ij}|\theta \right)=\pi_{o}f_{0}\left(Z_{ij}|\theta \right)+\pi_{1}f_{1}\left(Z_{ij}|\theta \right)$$

where $\pi_{0}$ is the mxining proportion of EE transcripts and $\pi_{1}=\left(1-\pi_{0}\right)$ is the proportion of DE transcripts,  θ is a vector parameters involved to characterize the distributions. Let *F_i _*be the minor allele frequency of the *i*th SNP then we model the distribution of *Z_ij _*as a mixture model of the form:

(3) $$\text{Pr}\left(Z_{ij}|F_{i}\right)\propto {\left[f_{0}\left(Z_{ij}|F_{i}\right)\right]}^{1-\delta_{ij}}{\left[f_{1}\left(Z_{ij}|F_{i}\right)\right]}^{\delta_{ij}}$$

where $f_{1}\left(.\right)$ denotes the distribution of *Z_ij _*for nonzero associations between $G_{i}$ and $T_{j}$ and $f_{0}\left(.\right)$ denotes the distribution of $Z_{ij}$ for the zero associations. $\delta_{ij}$ isdefined as

$$\delta_{ij}=\left\{\begin{array}{c} 1\;\;if\;\;nonzero\;\;association\;\;is\;\;present\\ 0\;\;if\;\;zero\;\;association\;\;is\;\;present \end{array}\right)$$

For any transcript and any SNP there may be three possible relations: no association, positive association and negative association. Extending the idea of two component mixture model, the distribution of the test statistics is modeled by the following mixture model:

(4) $$f\left(Z_{ij}|\psi_{i},F_{i}\right)=\sum_{k=0}^{2}\pi_{ik}f_{k}\left(Z_{ij};\mu_{k},\tau_{k}^{2},\nu_{k}\right)$$

Where

$$\psi_{i}=\left(\pi_{i}^{\prime },\theta_{i}^{\prime },\nu_{i}^{\prime }\right)$$

$$\pi_{i}=\left(\pi_{0i},\pi_{1i}\right)\;\theta_{i}=\left(\mu_{1i},\mu_{2i},\tau_{1i}^{2},\tau_{2i}^{2}\right)\;\nu_{i}=\left(\nu_{1i},\nu_{2i}\right)$$

with $\mu_{0i}=0,\tau_{0i}^{2}=1$. Mixing proportions $\pi_{ik}$ are nonnegative constantsand sum to one for fixed *i*. $f_{0}\left(.\right)$ corresponds to distribution for no associationwhereas $f_{1}\left(.\right)$ and $f_{2}\left(.\right)$ correspond to distributions related to positive and negativeassociation respectively. In a recent work, Noma and Matsui [27], have used semiparametric hierarchical mixture model where the distribution of mean expression level of a transcript is considered to be a three-component mixture distribution.

Full Bayesian analysis of (4) will require prior specifications of $\pi ,\theta ,\nu ,f_{0}\left(Z\right)$ and $f_{1}\left(Z\right)$. However, one can use the massively parallel structure of microarray data to estimate an empirical Bayes estimate of the posterior probability. These huge data motivates to be quite empirical rather than specifying *a-priori *models in favor of data-based investigations [27].

### Empirical Bayes, false discovery rates (fdr) and local false discovery rate (lfdr)

False discovery rate (fdr) is defined as the expected proportion of errors committed by falsely rejecting null hypotheses. Benjamini and Hochberg's [2]fdr criterion has very close relation with the empirical Bayes analysis. This relation improved the connection between Bayesian and frequentist testing theory. The close connection between fdr and the empirical Bayes methodology follows directly from Bayes theorem and this has been established by the "Equivalence theorem"[28]. Tail area rejection regions like $\left\{Z_{ij}<z\right\}$ are common in the frequentist framework. According to this theorem, if the tail area rejection region is taken to be as large as possible subject to the constraint that the estimated Bayes proportions of false discoveries is less than  α, then the frequentist expected proportion of false discoveries is also less than  α.

The empirical Bayes approach suggests a local version of the fdr called local false discovery rate (lfdr). The Bayes probability that a transcript $T_{j}$ for SNP $G_{i}$ is "EE" given the test statistic $Z_{ij}$, is known as $lfdr\left(Z_{ij}\right)$ and it is defined as

$$lfdr\left(Z_{ij}\right)\equiv \mathrm{Pr}\left(T_{j}\;\;is\;\;\text{EE}|Z_{ij}\right)=\pi_{i0}f_{0}\left(Z_{ij}\right)/f\left(Z_{ij}\right)$$

Analytically, fdr is a conditional expectation of lfdr defined as

$$fdr\left(Z_{ij}\right)=\overset{Z_{ij}}{\underset{-\infty }{\int }}lfdr\left(Z\right)f\left(Z\right)dZ/\overset{Z_{ij}}{\underset{-\infty }{\int }}f\left(Z\right)dZ=E_{f}\left\{lfdr\left(Z|Z\leq Z_{ij}\right)\right\}$$

For the above set up in (3), $1-\delta_{ij}$ represents the local false discovery rate (lfdr) and fdr can be estimated:

(5) $${\hat{\delta }}_{ij}=\frac{\pi_{i1}f_{1}\left(Z_{ij}\right)}{\left(1-\pi_{i0}\right)f_{0}\left(Z_{ij}\right)+\pi_{i1}f_{1}\left(Z_{ij}\right)};lfdr\left(Z_{ij}\right)=1-{\hat{\delta }}_{ij}$$

and hence

(6) $$fdr\left(Z_{ij}\right)=\frac{\left(1-\pi_{1i}\right)\int_{\left|Z_{ij}\right|}^{\infty }f_{0}\left(x\right)dx}{\left(1-\pi_{1i}\right)\int_{\left|Z_{ij}\right|}^{\infty }f_{0}\left(x\right)dx+\pi_{1i}\int_{\left|Z_{ij}\right|}^{\infty }f_{1}\left(x\right)dx}$$

$\nu_{0i}$ is estimated bypermutation method (Efron et *al*. [4]) and $p_{oi}$ is estimated from the nonnegative constraint

$$p_{0i}\leq \underset{Z}{\min}\frac{f_{i}\left(Z\right)}{f_{i0}\left(Z\right)}$$

All other parameters will be estimated by EM algorithm assuming $f_{i0}\left(.\right)$ to be known. There are some practical difficulties with the lfdr that relies on densities. The estimation of null becomes more problematic in the far tails. It is relatively easier to work with cumulative distribution function than work with densities. Identification of discoveries by lfdr may not be reproducible for a new data. Therefore, even in empirical Bayes framework, fdr should be preferred.

#### Nonparametric empirical Bayes (NPEB)

The main difference between parametric empirical Bayes (PEB) and nonparametric empirical Bayes (NPEB) is the way in which $f_{1}\left(.\right)\;\text{and}f_{2}\left(.\right)$ are treated. In PEB model, the functional form of $f_{1}\left(.\right)\;\text{and}f_{2}\left(.\right)$ are known, i.e., we have a parametric family of priors. In contrast, the NPEB does not assume the functional form to be known. Though NPEB methods are quite powerful, these are more suitable for large sample analyses. To compute the fdr under NPEB setup, we have followed the algorithm proposed by Efron et al. [4].

#### ECME algorithm

To fit a mixture model, EM algorithm is widely used. In case of *t *distribution the mean parameter  μ and variance component $\tau^{2}$ can easily be estimated by EM algorithm assuming that degrees of freedom  ν is known. However when  ν is unknown EM still can be used as demonstrated by Lange, Little and Taylor [29]. But this method appears to be very slow (Liu and Rubin [30]) and an extension has been proposed by Meng and Rubin [31] as ECM algorithm. This is a generalization of EM algorithm where the E step remains the same butthe M step is replaced by CM (constrained or conditional maximization) step. ECM algorithm is basically a generalized EM (GEM) as shown by Meng and Rubin [31]. Incidentally, the rate of convergence, in terms of iterations, for this ECM algorithm is slower compared to EM. To overcome this computational problem, Liu and Rubin [30] propose an efficient algorithm ECME which is again an extension of ECM algorithm. Though this is not a GEM, it converges faster.

For the  i -th SNP, the complete data is defined as

$$D_{iC}=\left(Z_{ij},\delta_{ijk1},\delta_{ijk2}\ldots \ldots \ldots .\delta_{ijkn},U_{i1},U_{i2}\ldots \ldots .U_{in}\right)$$

where

$$\delta_{ijks}=\left\{\begin{array}{c} 1\;\;if\;\;s\;\;th\;\;observation\;\;of\;\;Z_{ij}\in kth\;\;component\\ 0\;\;otherwise\;\;\;\;\;\;\;\;\;\;\;\;\;\;\;\;\;\;\;\;\;\;\;\;\;\;\;\;\;\;\;\;\;\;\;\;\;\;\;\;\;\;\;\;\;\;\;\;\;\;\;\;\;\;\;\;\;\;\;\;\;\;\; \end{array}\right)$$

and $U_{i}$ s are independently distributed gamma variables.

McLachlan and Krishnan [32] have already discussed the application of the EM algorithm for ML estimation in case of single component *t *distribution. In ECME algorithm, this result has been extended to cover the present set up of a 3-component mixture of *t *distribution. For the sake of brevity, in this section we omit the suffix *ij *for all the variables. To define *t *distribution with mean  μ, variance $\tau^{2}$ and degrees of freedom  ν, we proceed as follows:

$$If\;\;Z|U=u,\delta_{ks}=1\sim N\left(\mu ,\frac{\tau^{2}}{u}\right)and\;\;U\sim \Gamma \left(\frac{\nu }{2},\frac{\nu }{2}\right)$$

then marginally, $Z\sim t\left(\mu ,\tau^{2},\nu \right)$.

Following the above definition, the complete data likelihood $L_{iC}$ can be factorized a product of three terms-marginal densities of  δ s, the conditional densities of U|δ, and conditional densities of Z|U=u,δ. In notation, the log-likelihood of the complete-data can be expressed as

(7) $$\text{log}\;\;L_{C}\left(\psi \right)=\text{log}\;\;L_{1C}\left(\pi \right)+\text{log}\;\;L_{2C}\left(\nu \right)+\text{log}\;\;L_{3C}\left(\theta \right)$$

where

(8) $$\text{log}\;\;L_{1C}\left(\pi \right)=\sum_{\text{k}=0}^{2}.\sum_{\text{s}=1}^{\text{n}}\delta_{ks}\log\pi_{k}$$

(9) $$\text{log}\;\;L_{2C}\left(\nu \right)=\sum_{\text{k}=0}^{2}.\sum_{\text{s}=1}^{\text{n}}\delta_{ks}\left\{-\log\Gamma \left(\frac{\nu_{k}}{2}\right)+\frac{1}{2}\nu_{k}\log\Gamma \left(\frac{\nu_{\text{k}}}{2}\right)+\frac{1}{2}\nu_{k}\left(\log u_{s}-u_{s}\right)-\log u_{s}\right\}$$

and

(10) $$\text{log}\;\;L_{3C}\left(\theta \right)=\sum_{\text{k}=0}^{2}.\sum_{\text{s}=1}^{\text{n}}\delta_{ks}\left\{-\frac{1}{2}\pi_{k}\log\left(2\pi \right)-\frac{1}{2}\tau_{k}^{2}-\frac{1}{2}\frac{u_{s}{\left(z-\mu_{k}\right)}^{2}}{\tau_{k}^{2}}\right\}$$

#### E-Step

To compute the E-step of the proposed algorithm, at (t+1)th step we need to calculate $Q\left(\psi ;\psi^{\left(t\right)}\right)$, the current conditional expectation of the complete-data log likelihood function $\log L_{C}\left(\psi \right)$. From equation (4) to (7), we can write

(11) $$Q\left(\psi ;\psi^{\left(t\right)}\right)=Q_{1}\left(\pi ;\psi^{\left(t\right)}\right)+Q_{2}\left(\nu ;\psi^{\left(t\right)}\right)+Q_{3}\left(\theta ;\psi^{\left(t\right)}\right)$$

where

(12) $$Q_{1}\left(\pi ;\psi^{\left(t\right)}\right)=\sum_{k=0}^{2}.\sum_{s=1}^{n}E_{\psi^{\left(t\right)}}\left(\delta_{ks}\text{|}z_{s}\right)=\sum_{k=0}^{2}.\sum_{s=1}^{n}\xi_{ks}^{\left(t\right)}\log\pi_{k}$$

and

(13) $$\xi_{ks}^{\left(t\right)}=\frac{p_{k}^{\left(\text{t}\right)}f\left(Z_{s};\mu_{k}^{\left(t+1\right)},{\tau^{2}}_{k}^{\left(t+1\right)},\nu_{k}^{\left(t+1\right)}\right)}{f\left(Z;\psi^{\left(t+1\right)}\right)}$$

which is the posterior probability that  Z belongs to the k-th component of the mixture based on current fit $\psi^{\left(\text{t}\right)}$.

Similarly,

(14) $$Q_{2}\left(\nu ;\psi^{\left(t\right)}\right)=\overset{2}{\underset{k=0}{\sum }}.\overset{n}{\underset{s=1}{\sum }}\xi_{ks}^{\left(t\right)}\left[-\log\Gamma \left(\frac{\nu_{k}}{2}\right)+\frac{1}{2}\nu_{k}\log\Gamma \left(\frac{\nu_{\text{k}}}{2}\right)+\frac{1}{2}\nu_{k}\left\{\overset{n}{\underset{s=1}{\sum }}\left(\log{u_{ks}}^{\left(t\right)}-u_{ks}^{\left(t\right)}\right)+\psi \left(\frac{\nu_{\text{k}}^{\left(\text{t}\right)}+1}{2}\right)-\log\left(\frac{\nu_{\text{k}}^{\left(\text{t}\right)}+1}{2}\right)\right\}\right]$$

Where

(15) $$u_{ks}^{\left(t\right)}=\frac{\nu_{k}^{\left(t\right)}+1}{v_{k}^{\left(t\right)}+{\left(Z_{s}-\mu_{k}^{\left(t\right)}\right)}^{2}/\tau_{k}^{\left(t\right)}}$$

ψ(.) is a digamma function and

(16) $$Q_{3}(\theta ;\psi^{\left(t\right)})=\overset{k=0}{\underset{2}{\sum^{\text{​}}}}.\overset{s=1}{\underset{n}{\sum^{\text{​}}}}\xi_{ks}^{\left(t\right)}[-\frac{1}{12}\log(2\pi )+\frac{1}{12}\log u_{ks}{}^{\left(t\right)}-\frac{1}{12}u_{ks}^{\left(t\right)}{\left((Z_{s}-\mu_{k})/\tau_{k}\right)}^{2}\}]$$

#### CM-step

In usual M-step parameters  π,  ν,  θ can be estimated by considering equations (10) - (12) independently. The new updates for π,θ can be obtained as a closed form solution whereas for  ν an iterative procedure may be used using the following equations:

(17) $$p_{k}^{\left(t+1\right)}=\overset{n}{\underset{s=1}{\sum }}\frac{\xi_{ks}^{\left(t\right)}}{n}$$

(18) $$\mu_{\text{k}}^{\left(t+1\right)}=\left(\sum_{s=1}^{n}\xi_{ks}^{\left(t\right)}u_{ks}^{\left(t\right)}Z_{s}\right)/\sum_{s=1}^{n}\xi_{ks}^{\left(t\right)}u_{ks}^{\left(t\right)}$$

(19) $$\tau_{\text{k}}^{\left(t+1\right)}=\sum_{s=1}^{n}\xi_{ks}^{\left(t\right)}u_{ks}^{\left(t\right)}{\left(\frac{Z_{s}-\mu_{k}}{\tau_{k}}\right)}^{2}/\sum_{s=1}^{n}\xi_{ks}^{\left(t\right)}$$

and $\nu_{\text{k}}^{\left(t+1\right)}$ is the solution of the following equation

(20) $$\{-\psi \left(\frac{\nu_{\text{k}}}{2}\right)+\log\left(\frac{\nu_{\text{k}}}{2}\right)+1+\frac{1}{n_{k}^{\left(t\right)}}\sum_{s=1}^{n}\xi_{ks}^{\left(t\right)}\log\Gamma \left(\frac{\nu_{\text{k}}}{2}\right)+\frac{1}{2}\nu_{k}\left\{\sum_{s=1}^{n}\left(\log{u_{ks}}^{\left(t\right)}-u_{ks}^{\left(t\right)}\right)+\psi \left(\frac{\nu_{\text{k}}^{\left(\text{t}\right)}+1}{2}\right)-\log\left(\frac{\nu_{\text{k}}^{\left(\text{t}\right)}+1}{2}\right)\right\}=0$$

To get an efficient algorithm, let us partition  ψ as $(\psi_{1}^{'},\psi_{2}^{'})'$ where $\psi_{1}$ contains all the parameters except parameters corresponding to degree of freedom of t-distributions. The above M-step is replaced by two CM-steps, as follows.

**CM-Step 1**. Keeping $\psi_{2}$ fixed, i.e.  ν is fixed at $\nu^{\left(\text{t}\right)}$, maximize $\text{Q}\left(\psi ;\psi^{\left(\text{t}\right)}\right)$ to get $\psi_{1}^{\left(t+1\right)}$

**CM-Step 2**. Now fix $\psi_{1}$ at $\psi_{1}^{\left(t+1\right)}$ and calculate $\psi_{2}^{\left(t+1\right)}$ by maximizing $\text{Q}\left(\psi ;\psi^{\left(\text{t}\right)}\right)$

Furthermore to make the algorithm more efficient, after the first CM-step, we replace the E-step with $\psi =(\psi_{1}^{\left(t+1\right)}{}^{'},\psi_{2}^{\left(t\right)}{}^{'})'$ instead of $\psi =(\psi_{1}^{\left(t\right)}{}^{'},\psi_{2}^{\left(t\right)}{}^{'})'$.

### Simulation study

To assess the proposed methodology, a small sample simulation study has been performed. This gives an idea whether or not the parameters are well estimated and most importantly, they provide information of false discovery rates.

**Figure 1:**
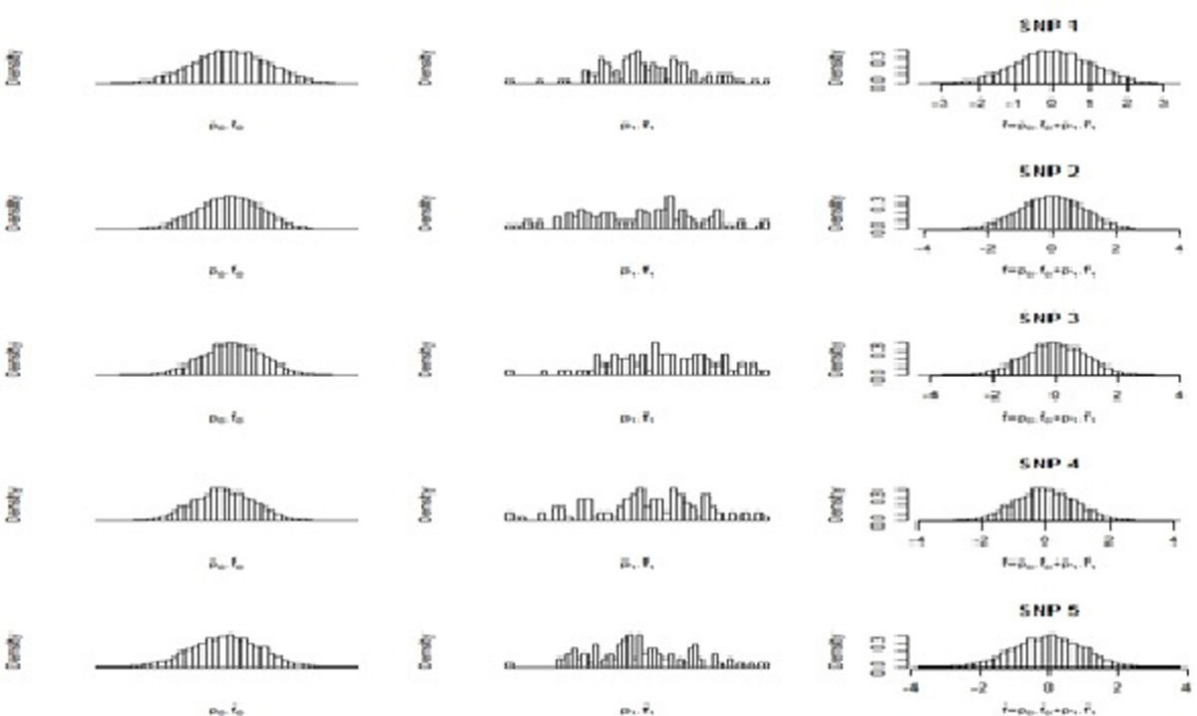
**A part of the simulated data for **$p_{1}=0.05$.

First we simulated a dominant model with 10,000 transcripts and 10 SNPs. The equivalently expressed (EE) transcripts are generated from N(0,1) after log-transformation. We have simulated the data under three choices of proportions of differentially expressed (DE) transcripts ($p_{1}$). We have taken $p_{1}$ to be (0.01, 0.05, 0.10). If the transcript is DE, it has to be generated from N(4,0.5) after log-transformation. The controlled fdr are also assumed to be (0.01, 0.05, 0.10) for these data sets. For $p_{1}=0.05$, the simulated data is given in Figure 1.

**Figure 2:**
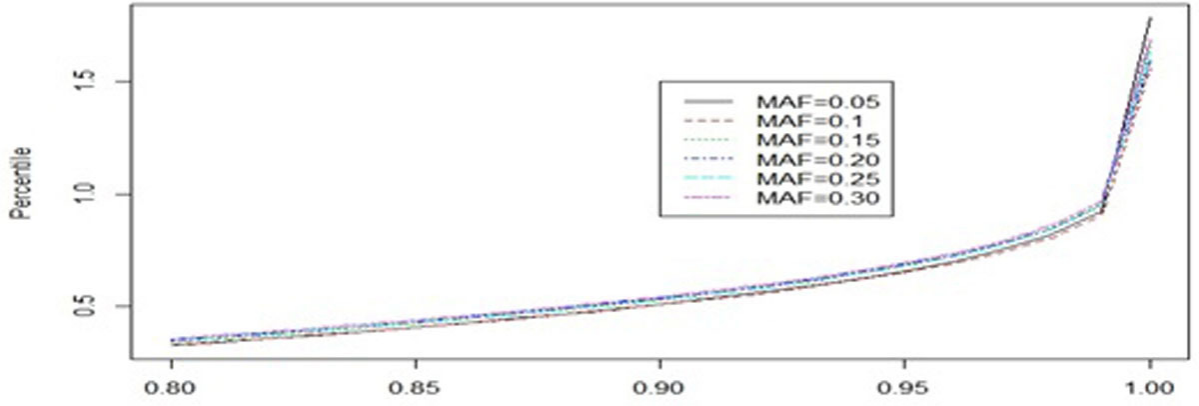
**Effect of minor allele frequency (MAF) on the null distribution**. Only upper quantiles (from 80%) have been considered as lower quantiles showing almost no difference.

The impact of minor allele frequency (MAF) on the distributions under null has also been studied. Under null, for a t-distribution, the only parameter to be estimated is its degrees of freedom. The comparison has been made by computing different quantiles for six choices of MAFs. For the lower quantiles, they almost overlapped with each other. Very small deviations are observed for upper quantiles (Figure 2).

**Figure 3:**
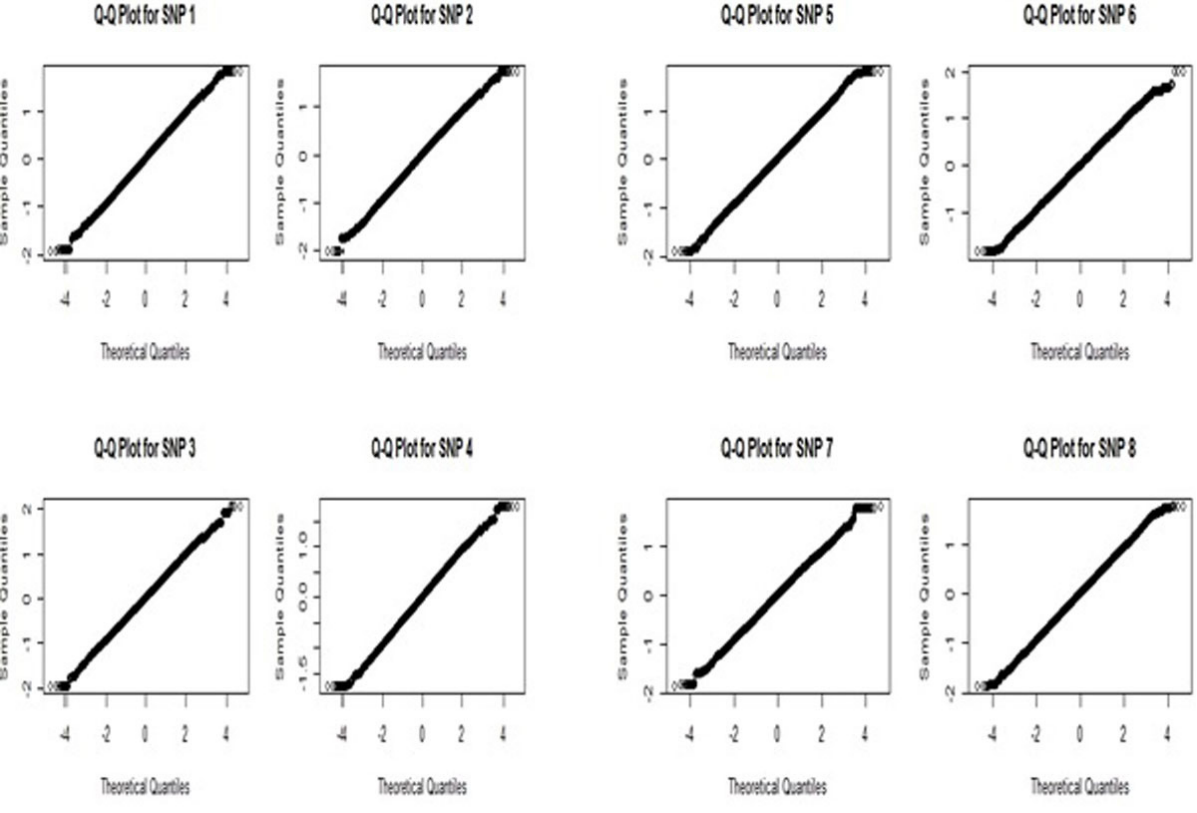
**QQ-plot for eight SNPs**.

For the 10 SNPs, we fitted the null distribution using permutation method in a balanced way. From each group, randomly selecterd 35 samples are shifted from one group to the other and the value of the statistic is noted. This process is repeated 40 times and histograms are plotted. From the histograms, the degrees of freedom corresponding to the null distribution for eack SNP is estimates. To get an idea about the goodness-of-fit, Q-Q plots are done (Figure 3). These plots show that the null distribution is well approximated by the standardized t-distribution with appropriate degrees of freedom.

Parameters related to the mixture model (4) are estimated using proposed ECME algorithm after estimating the null distribution using permutation method. Then FDR is computed under both proposed parametric empirical Bayes and nonparmetic empirical Bayes setup and the result is given in Table 1.

**Table 1:** The True FDR Performance of Controlled FDR in EB Models

**True fraction of DE**	**Controlled FDR**					
	
	**Nonparametric empirical Bayes**			**Parametric empirical Bayes**		
	
	0.01	0.05	0.10	0.01	0.05	0.10
0.01	0.004	0.029	0.067	0.005	0.042	0.090
0.05	0.006	0.041	0.079	0.006	0.045	0.094
0.10	0.007	0.043	0.087	0.008	0.047	0.097

It is evident from the above table that the nonparmateric empirical Bayes is much conservative compared to its parametric alternative. For parametric set up, the true FDR is very much close to the controlled one, whereas, for nonparametric empirical Bayes these values are not so close as the true fraction of DE transcripts increases.

### HLC data analysis

We applied the empirical Bayes model to analyze a sequencing data publicly available. In the current study, we have started with liver tissue data of 213 Caucasian samples from apreviously described human liver cohort (LHC) (Yang et al. [33]). To get the genotypes and gene expression profiles, DNA and RNA have been isolated. Illumina platform is used to get the expressions. After putting some filtration (MAF>5%, HWE<10^-5^,) we are left with 173 samples, 472,000 SNPs and 30,000 expressions.

**Figure 4:**
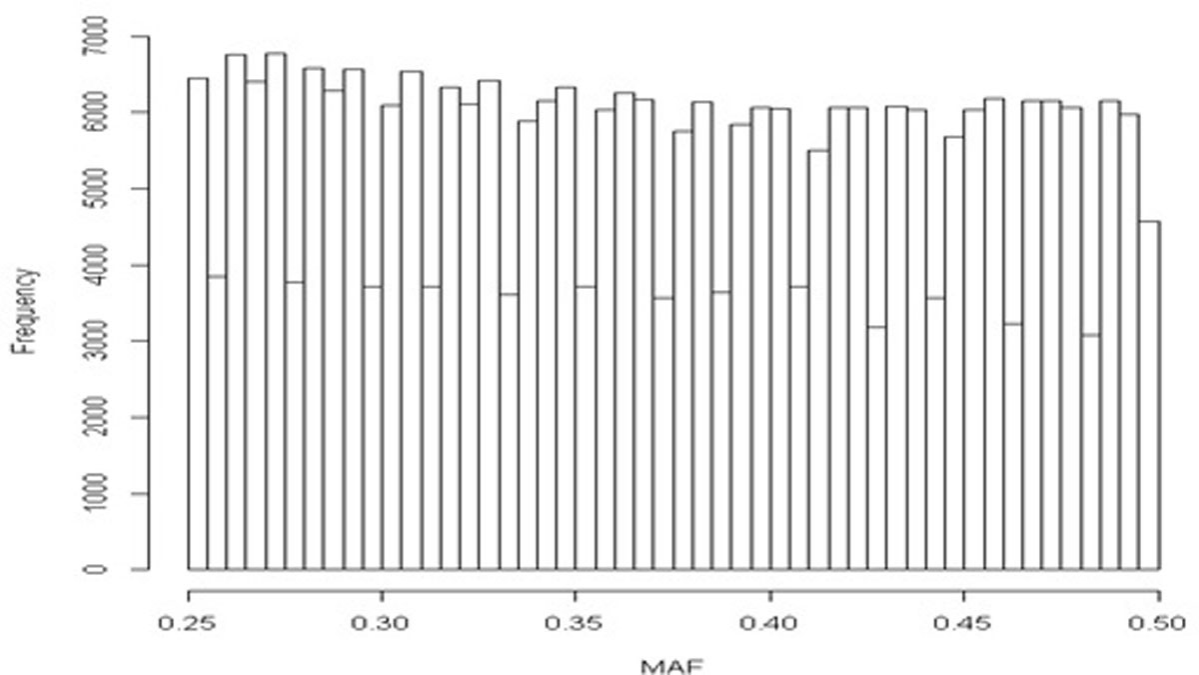
**Minor allele frequency (MAF) distribution**. X axis corresponds to minor allele frequency 25% to 50%.

The distribution of minor allele frequency (MAF) over SNPs is given in the histogram (Figure 4). For all possible SNP-transcript combinations, test statistic, $Z_{ij}$ s are computed. We fit the mixture model using the ECME algorithm in R 2.15.1 after estimating the null distribution using permutation method. However, due to high dimension data, it becomes very difficult to fit a mixture model using the proposed algorithm. For the sake of parsimony, we further filtered the data and ECME algorithm is used for only top SNPs with $p-value<10^{-3}$. For these top SNPs, the mixture model is fitted and estimates are obtained. To compute lfdr and FDR from (5) and (6) respectively, these estimates are used.

## Conclusion

To compare our result with [33], we focus on 18 of the 54 P450 genes used in the study. These are CYP3A5, CYP2D6, CYP4F12, CYP2E1, CYP2U1, CYP1B1, CYP2C18, CYP4F11, CYP4V2, CYP2F1, CYP39A1, CYP26C1, CYP2C19, CYP2C9, CYP2S1, CYP46A1, CYP4A11 and CYP4X1.However our method fails to identify a single SNP with FDR<10% for CYP2R1 and that gene symbol has been excluded from the table (Table 2). It can be seen from the table (Table 2) that for a threshold of 10% FDR number of significant eQTL pairsis4916.Since we have considered only top SNPs, this may be an overestimate. SNPs which are within <1-Mb distance from gene location are defined as cis-SNPs. It is interesting to note that, among these 18 genes, the first five (CYP3A5, CYP2D6, CYP4F12, CYP2E1 and CYP2U1) having more than 40 cis-SNPs. In all cases FDR based analysis results in identifying more cis-SNPs for these 18 genes compared to that of Yang et al. (2010) [33].

**Table 2:** Number of eQTL pairs after crossing the threshold of FDR

Gene symbol	No. of SNPs (FDR<10%)	No. of cis-SNP	No. of cis-eSNP (FDR<10%) by Yang et al. (2010)
CYP3A5	263	62	56
CYP2D6	264	67	54
CYP4F12	392	55	46
CYP2E1	130	45	31
CYP2U1	549	45	26
CYP1B1	168	21	13
CYP2C18	90	13	9
CYP4F11	169	15	7
CYP4V2	159	25	3
CYP2F1	324	10	2
CYP39A1	448	17	2
CYP26C1	154	29	1
CYP2C19	356	7	1
CYP2C9	413	20	1
CYP2S1	319	10	1
CYP46A1	430	7	1
CYP4A11	461	4	1
CYP4X1	151	3	1

## Discussion

In contrast to previously available methods based on p-values, the empirical Bayes method uses local false discovery rate (lfdr) as the threshold. This method controls false positive rate. For a particular SNP, the lfdr is computed for the site-specific evidence whereas the FDR averages over other sites with stronger evidence. There are some limitations of using FDR which may result in misleading inferences in genome studies. In such a situation, it is better to use lfdr which is a bit difficult to estimate compared to FDR.However there is still one computational problem which needs much attention. Due to the high dimensionality in the data, sometimes existing algorithms fail. This necessitates the need to find some more efficient algorithms. The choice of threshold FDR value is an important deciding factor in such studies. It would be interesting to see, how number of cis-SNPs vary with the change in FDR threshold. In this way FDR criterion can be used to estimate number of SNPs that we may need to consider.

## Competing interests

The authors declare that they have no competing interests.

## Declarations

The publication costs were funded by the authors through P50 CA113001 (Huang, T.M.), R01 GM088076 (Skaar, T.), R01 HS019818 (Dexter).

This article has been published as part of *BMC Genomics *Volume 14 Supplement 8, 2013: Selected articles from the International Conference on Intelligent Biology and Medicine (ICIBM 2013): Genomics. The full contents of the supplement are available online at http://www.biomedcentral.com/bmcgenomics/supplements/14/S8.
